# Intelligent Transportation Using Wireless Sensor Networks Blockchain and License Plate Recognition

**DOI:** 10.3390/s23052670

**Published:** 2023-02-28

**Authors:** Fares Alharbi, Mohammed Zakariah, Reem Alshahrani, Ashwag Albakri, Wattana Viriyasitavat, Abdulrahman Abdullah Alghamdi

**Affiliations:** 1 Department of Computer Science, College of Computing and IT, Shaqra University, Shaqra 15526, Saudi Arabia; 2 College of Computer and Information Science, King Saud University, Riyadh 11442, Saudi Arabia; 3 Department of Computer Science, College of Computers and IT, Taif University, P.O. Box 11099, Taif 21944, Saudi Arabia; 4 Department of Computer Science College of Computer Science & Information Technology, Jazan University, Jazan 45142, Saudi Arabia; 5 Chulalongkorn Business School, Faculty of Commerce and Accountancy, Chulalongkorn University, Bangkok 10330, Thailand

**Keywords:** intelligent transportation system, license plate recognition, blockchain technology, privacy in ITS, internet of vehicles

## Abstract

License Plate Recognition (LPR) is essential for the Internet of Vehicles (IoV) since license plates are a necessary characteristic for distinguishing vehicles for traffic management. As the number of vehicles on the road continues to grow, managing and controlling traffic has become increasingly complex. Large cities in particular face significant challenges, including concerns around privacy and the consumption of resources. To address these issues, the development of automatic LPR technology within the IoV has emerged as a critical area of research. By detecting and recognizing license plates on roadways, LPR can significantly enhance management and control of the transportation system. However, implementing LPR within automated transportation systems requires careful consideration of privacy and trust issues, particularly in relation to the collection and use of sensitive data. This study recommends a blockchain-based approach for IoV privacy security that makes use of LPR. A system handles the registration of a user’s license plate directly on the blockchain, avoiding the gateway. The database controller may crash as the number of vehicles in the system rises. This paper proposes a privacy protection system for the IoV using license plate recognition based on blockchain. When a license plate is captured by the LPR system, the captured image is sent to the gateway responsible for managing all communications. When the user requires the license plate, the registration is done by a system connected directly to the blockchain, without going through the gateway. Moreover, in the traditional IoV system, the central authority has full authority to manage the binding of vehicle identity and public key. As the number of vehicles increases in the system, it may cause the central server to crash. Key revocation is the process in which the blockchain system analyses the behaviour of vehicles to judge malicious users and revoke their public keys.

## 1. Introduction

One of the essential components in the development of an intelligent digital city is the intelligent transportation system (ITS). The growth of intelligent transportation contributes to improving public safety and propels the blockchain sector. The unique identifier on the roads and in public transit for the IoV is the information from the license plate. In 2021, the number of vehicles expanded, and this resulted in new challenges and issues for the management and control of automobiles in the transportation system, including privacy concerns and significant resource consumption in large cities [1]. The study of automatic LPR in the IoV to enhance service to management by detecting and recognising vehicle license plates on roadways is therefore of major research significance and practical usefulness. In automated transportation systems, privacy and trust are important challenges. Privacy issues about data collection have been brought up by the use of LPR systems [2,3]. Vehicles that are or are not under criminal investigation are not differentiated by LPR systems. In nations where the storing of personal data without permission or justification allowed by law is protected, this function is in violation of data protection regulations. Organizations that use LPR systems have been questioned by individuals and groups that protect individual privacy.

A vehicle’s license plate, location, date, and time of the image’s capture, as well as the image itself, can be used to infer information about the driver [4]. Therefore, it is evident that privacy protection strategies are required in cities that employ LPR devices for car monitoring. The privacy of the data that is collected must be considered while implementing these systems, and current data protection rules must not be broken in order to retain and handle that data, based on the reference publications [2,5,6], where the researchers found a lack of privacy protections for LPR systems. A storage architecture was proposed in [7] that utilizes blockchain-based technology to address the privacy gap and ensure data confidentiality for LPR platforms. This kind of organization is based on the blockchain platform, a decentralized network that can carry out smart contracts. Each smart contract in this concept adheres to the privacy preferences of a license plate that has been publicly encrypted for anonymity. As a result, if data security is set up in the smart contract connected to the license plate, the data that the LPR technology has retrieved cannot be stored. A gateway in this architecture allows blockchain smart contract authorization. A responsible user might update the smart contract associated with a specific license plate in situations when incentive is necessary by legislation to justify the protection of the data collected by the LPR system.

**Figure 1:**
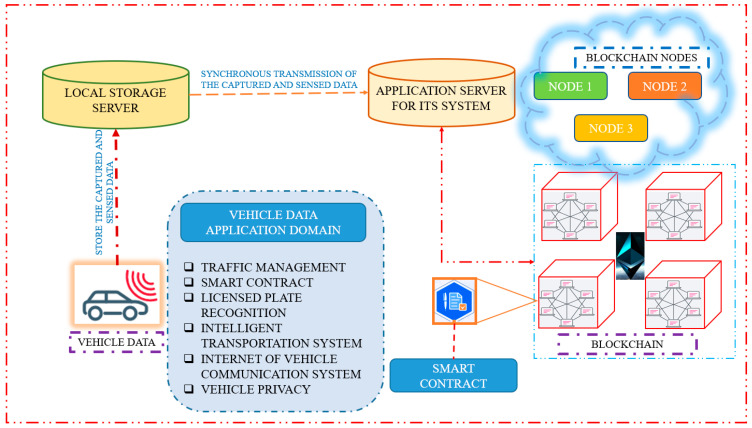
Design of an intelligent transportation management system using blockchain and smart contracts.

The architecture of an ITS management system that recognizes license plates using blockchain and smart contracts is shown in Figure 1, wherein vehicle data is transmitted to the local storage server before going to the ITS application server. Traffic management, smart contracts, LPR, and vehicle privacy are the components of the vehicle data application domain.

The concept of vehicle ad hoc networks (VANETS), that evolved from the Internet of Thing (IoT), is at the heart of autonomous driving technologies [8]. The IoV system, which integrates cutting-edge communication technology, has two basic communication components: vehicular mobile internet [8,9] and intervehicular communication. A distributed, decentralised database system is precisely what blockchain is. It supports the immutable, continuously growing blockchain structure of data blocks between participating nodes [8]. Each block contains transactions that are linked to the Merkle root, and each block may be logically separated into a blockhead and a block body. In the blockhead, a hash value is connected with each block. The most recent transaction data will be kept by each client, creating a decentralised storage system. The blockchain will then use the consensus mechanism to synchronise the transaction information throughout the entire network. Even if some nodes fail, the scheme will still work [10]. At the service level, privacy and trust remain a little fuzzy, despite some discussion of security, privacy, and trust at the vehicle network level in [5,11]. The suggested IoV [1] services in the literature make use of personally identifiable information, which might include data which was unintentionally gathered from third parties (such as images of pedestrians or private properties). Both users and non-users may experience privacy violations as a result of such negligent information sharing. There are still further concerns regarding information access via the IoV, such as whether it can be used to spy on people’s activities. However, privacy can be a complicated issue because restricting the transmission of information might, in some cases, have a negative influence on services and confidence, making it difficult to demonstrate the reliability of service providers and the IoV.

To address IoT privacy and security concerns, a proposed solution is to combine commuters and public transportation into a spectrum of transportation. This approach aims to develop a secure reputation system, allowing users to input traffic reports and access accurate trip information using various blockchain technologies [12]. The resident’s activities and prior interactions with the type of data presented to the system would establish the reputation. The system can advise users of optimal pathways based on the traffic situations in the locations from which the data is being transmitted and the user’s desired method of transportation. Both reputation and actual traffic data are permanently stored on a tamper-proof blockchain that cannot be altered by attackers [12,13]. Users within the same cluster or region determine the accuracy of the information using a consensus method that takes participants’ credentials into account. Blockchains set up peer-to-peer networks that let users employ decentralized peer-to-peer consensus protocols to continuously validate interactions. Many people assume of blockchains as unbreakable, time-stamped database systems. A variety of studies [14,15,16], and industrial viewpoints, have been gathered to document the possible disruptive effects of this rising technology trend [15]. 

In response to the likelihood of privacy and security vulnerabilities increasing, the centralized Machine Learning (ML) paradigm is transitioning to a more distributed and decentralized learning framework, specifically in a Federated Learning (FL) context. To minimize privacy threats, Google introduced FL [5], which comprises a group of people learning a model collectively. The central server employs many clients, commonly known as workers, to help prepare a Deep Neural Network (DNN) model. The central server offers the clients with an initial training model. Each client computes local updates to the global model using this model and its own local dataset, such as stochastic gradient descent. After a specified training period, each sender transmits their own improvements to the central server. To establish a global model, the central organization integrates the local models. But until a realistic general model is established, these processes are repeated. As a result, data privacy is guaranteed because clients do not send and keep local datasets at the main server. Compared to the conventional decentralized learning methodologies, FL is anticipated to offer additional benefits.

Despite the fact that FL has great security for learning structures, it actually relies on a centralised aggregator. In order to draw mobile devices to the training process, an economic model is also necessary. A malicious vehicle may once again result in a poisoning attack and change data. Again, a self-centred vehicle might not be able to accept information assortment, resulting in the incorrect weights for a local model. In order to provide a decentralised system for managing incentives and firmly ensuring security and protection, blockchain is being used with FL in light of the likelihood of such potential attacks [17,18,19]. This article’s goal is to evaluate the benefits of smart contracts’ LPR-based intelligent transportation.

**Figure 2:**
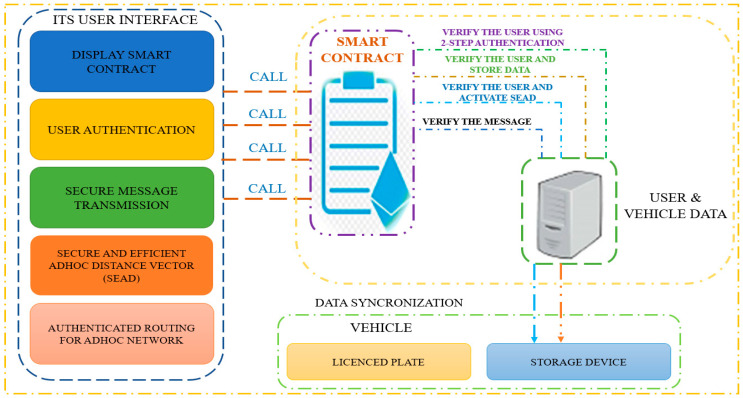
Proposed framework for ITS user interface.

Figure 2 illustrates the proposed framework for the Intelligent Transportation System (ITS) user interface, vehicle license plate, and user and vehicle data. The framework comprises five user interfaces, namely user authentication, secure message transmission, secure and efficient ad hoc distance vector, authentic routing for ad hoc network, and smart contract interfaces. The user authentication interface is responsible for authenticating the user, which is crucial for ensuring secure access to the system. The secure message transmission interface ensures that messages between different entities within the system are transmitted in a secure and encrypted manner, preventing unauthorized access or interception. The secure and efficient ad hoc distance vector interface is designed to facilitate efficient communication between vehicles in the ad hoc network. The authentic routing for the ad hoc network interface ensures that only authorized and legitimate vehicles are allowed to participate in the ad hoc network, which enhances the overall security of the system. The smart contract interface is responsible for executing pre-defined conditions and rules for the transactions carried out on the system. This interface ensures that transactions are transparent, secure, and efficient, and eliminates the need for intermediaries, thus reducing transaction costs and enhancing user trust in the system. 

The following are this paper’s major contributions:

- This study explores the potential of blockchain technology and smart transportation systems that use a framework for recognizing license plates along with IoV applications.
- This paper explores the design and implementation of a license plate detection system utilizing blockchain technology, with a particular emphasis on resolving privacy and security issues.
- The proposed model was evaluated through a series of comprehensive experiments, which revealed that the detection and identification models outperformed existing approaches in terms of accuracy and speed, particularly in challenging real-world environments.

The remainder of this paper is structured as follows: In Section 2 of the literature review, existing methods are introduced. The details of the architecture are provided in Section 3. The methodology of the system is examined in Section 4 of this study. Findings and Discussion represent Section 5 and Section 6, respectively. This study, in Section 7, ends with some conclusions.

## 2. Literature Review

For an intelligent transportation system that makes use of LPR, a lot of work has been conducted in the field of blockchain technology. Additionally, anonymity is included to safeguard the blockchain-based application. In VANETs, privacy and trust management are significant issues since these networks broadcast messages that reflect traffic occurrences that should be approved by drivers nearby and repeated or deleted if the trust value is below a certain level. This discussion will also cover data protection regulations that safeguard individuals’ privacy during the storage and processing of personal data, illustrating how LPR systems undermine this privacy. According to Barrero et al. [2], the LPR system is an automatic image processing system that recognises a vehicle’s license plate number. These kinds of systems work using one or more images that cameras have captured, which could be in colour or white and black [2]. To retrieve the license identifier, the LPR system utilizes a variety of methods such as image processing, object detection, and pattern recognition. After obtaining the license plate number, it may be connected to data stored in databases, enabling for further exploration for a variety of reasons. Electronic payment systems (for parking and toll fees) are just a couple of examples. Other ones include monitoring systems that gauge traffic flow, surveillance, and car- and human-tracking [2]. The identification of the LPR system for a vehicle that also recognises its license plate number was the focus of studies by Fareed et al. [6]. This number is put up against interesting vehicle database records in criminal investigations. A law enforcement officer can stop a suspect vehicle once it has been identified, inspect it for evidence, and, if necessary, make arrests. When a vehicle’s license plate is photographed by a camera, even the license plate numbers of non-suspect vehicles are stored. Intrusion detection is a trendy topic with numerous significant applications, claim Shen et al. in their study [10]. They have the necessary tools and detection techniques for this. The security needs that should be considered when developing a secure architecture for VANET include authentication, integrity, accountability, non-repudiation, restricted credential usage, credential revocation, and data consistency [5,11]. 

A cascade license plate classifier based on colour saliency features was recommended by Bansal et al. [12] to recognise the genuine license plates in candidate zones. The license plate locating method also uses texture features since the license plate area’s pixel texture distribution is consistent. Due to a number of issues, including the complex environment and erratic lighting in the photos, the previous LPD approaches based on visual aspects have reached a development bottleneck. In terms of object detection, deep learning performs quite well. Character segmentation and character recognition are two of the LPR’s two steps that Zhang et al. [20] proposed. The SIFT feature, the projection algorithm, and extreme area extraction are typical methods for character segmentation. The blurring, noise, and deformation of character directions in the images are easily influenced by these methods, which directly causes recognition problems, and thus causes an issue with segmentation deviation. In addition, a few more approaches to license plate character recognition without character segmentation are also put forth. According to Sestrem et al. [21], the LPR system might identify a car’s license plate number upon detection and match it to entries in a vehicle database that are relevant to criminal investigations. Furthermore, a camera even records the license plate information of passing non-suspect automobiles. A smart contract is triggered by a transaction being addressed to it, in accordance with Devetsikiotis et al. [22]. The authors contend that smart contracts make it possible to manage data-driven interactions between entities in a network by defining immutable standards of connection. Due to the irrevocable nature of contracts on the Ethereum blockchain, it is impossible for unauthorised users to change the code once it has been written. The LPR system of a city may hold a lot of data due to its ability to recognise and record hundreds of license plate numbers per minute. The executable logic that operates on the blockchain network is known as smart contracts. Autonomy and decentralisation are two of smart contracts’ key characteristics. The Ethereum Virtual Machine writes, compiles, and generates byte code for smart contracts in certain programming languages (EVM). Dapps, or decentralised applications, are programmes that operate on the Ethereum blockchain [23].

Using blockchain technology in a novel way to protect data privacy and eliminate collision attacks is outlined in [24]. As a trusted source or proxy server, the blockchain network is used. Its job is to generate pairs of private and public keys, authenticate users, and monitor access to cloud-stored data in compliance with the encryption algorithm and associated data security. Moreover, if more users want to access the data, it must be re-encrypted. A contract that confirms the use of encrypted data stored on a cloud server can likewise be made using the blockchain network. Singh et al. offer, in [25], a communication strategy that lends legitimacy to reward-based automobile behaviour. Every vehicle in the system has information about it stored on the blockchain, such as reputation or public keys. These are uniquely identified by an encryption number that has been issued by reputable organisations like licensed dealers or car vendors. 

Yang et al.’s [26] presentation of a trust environment based on an intelligent vehicle architecture is another example. The three main elements of the proposed system are mobile cloud computing, communication network, and blockchain technology. The seven-layer blockchain design includes mechanisms for trust and privacy. The authors of [27] present a reputation system using the blockchain in which messages are rated by vehicles upon their observations. The system is made up of four separate types of entities: miners, common cars, evil cars, and reliable authorities in charge of vehicle registration and certification of capability. The vehicles are equipped with the ability to send traffic data, verify the legitimacy of messages received using the issuer’s reputation, and generate a rating. From among the cars, miners are picked to construct a block, holding all the ratings and deliver it to every vehicle. If the block is approved, it is examined and uploaded to the blockchain. Only ratings that have been verified by other traffic participants are stored on the blockchain. The system that is being offered neither saves traffic data nor offers privacy-preserving solutions. The summary of the works are as shown in below Table 1. 

This literature review discusses the use of LPR in ITS and its connection to blockchain technology. The review highlights issues of privacy, trust management, and data protection regulations in the use of LPR systems. The review covers a variety of approaches and techniques used in LPR systems, including character segmentation and character recognition. Furthermore, the review mentions the use of smart contracts to manage data-driven interactions between entities in a network, and blockchain technology to protect data privacy and eliminate collision attacks. The research gaps in the existing literature include a lack of discussion on the impact of LPR systems on society and the need for more research into privacy and security concerns in LPR systems. There is also a need for more research into the use of blockchain technology in the management of LPR systems, including the implementation of a decentralized system. To overcome these limitations, this study recommends a blockchain-based approach for IoV privacy security that makes use of LPR. A system handles the registration of a user’s license plate directly on the blockchain, avoiding the gateway. The detailed description of the proposed methodology is presented in the subsequent sections.

## 3. Architecture

When designing blockchain-based ITS designs, security and privacy must be considered as crucial considerations. Considering this, it was decided to utilize various technologies, including blockchain, smart contracts, encryption, anonymization, and others, to ensure a high level of privacy for users. The ITS architecture is presented in this section.

### 3.1. Smart Contracts and Encryption Technique

Smart contracts are used in the proposed design to store a user’s privacy preferences, including whether or not they want to be tracked [27]. The contract’s privacy options have been changed to permit tracking of license plates if it becomes necessary to keep track of a user.

Because of its high level of privacy and reduced block size as compared to other encryption techniques, elliptic-curve cryptography (ECC), a key-based approach for data encryption, has gained popularity [28]. The ECC is a type of public-key cryptography used for secure communication over the internet. It is based on the mathematics of elliptic curves, which are a type of algebraic curve. In ECC, each user has a public key and a private key. The public key can be shared with anyone, while the private key is kept secret. ECC offers the same level of security as traditional public-key cryptography, but with smaller key sizes, making it more efficient for use in resource-constrained environments such as mobile devices and smart cards. ECC is used in a variety of applications, including secure web browsing, digital signatures, and secure messaging. 

The cryptographic keys were created using ECC, and each user of the system acquired a set of private and public keys. The public key is used to protect and encode legal documents. Private and public keys are required for any federal agency that wants to track a user, with the public key being employed to authenticate who is authorized to access the database of LP.

### 3.2. Anonymity

All users will become anonymous as a result of the use of public keys, which also functions as a codename in terms of anonymity. This technique is used because, regardless of the fact that a public key is distributed across the network, only the holder of a particular license plate would be aware of the key associated with it. Particularly, users who are being monitored do not interact with one another.

### 3.3. Blockchain

To reduce the current processing burden, a private blockchain was chosen. Smart contracts ensure the immutability of the system, even with this type of blockchain, so that the contract terms can never be altered. It should be emphasized that the blockchain will only be accessed by the authorities [29]. In this method, the user will be notified that they are being followed through a notification sent by the gateway at the right interval.

### 3.4. Architecture Setup

The architecture was developed by taking into account the following three requirements, using the provided data:

- Unless the government obtains a legal order authorising it, it will not monitor someone who objects to being watched.
- A person can be monitored by the government. He or she should be informed about this surveillance at the conclusion of the investigation.
- If necessary, someone can be watched and made aware of that (e.g., monitoring of people on probation).

### 3.5. The Proposed Architecture for This Work Is Given Below

- The user must select the security settings for monitoring and have connection to the vehicle’s license plate. This data is recorded in a smart contract kept on a personal blockchain. The user’s private and public keys are also created at this stage. When a user needs a license plate, the registration is handled directly on the blockchain, bypassing the gateway, via a system. Additionally, if a smart contract is registered, only the authorised addresses can modify its privacy settings.
- The image of the license plate is delivered to the gateway in charge of handling all communications when it is recorded by an LPR system [3,29].
- The gateway records the image of this license plate in a storage service if the data protection option of the license plate permits image capturing. On the contrary, if the user makes a decision that prevents the capture of the plate, no image is stored [3].

**Figure 3:**
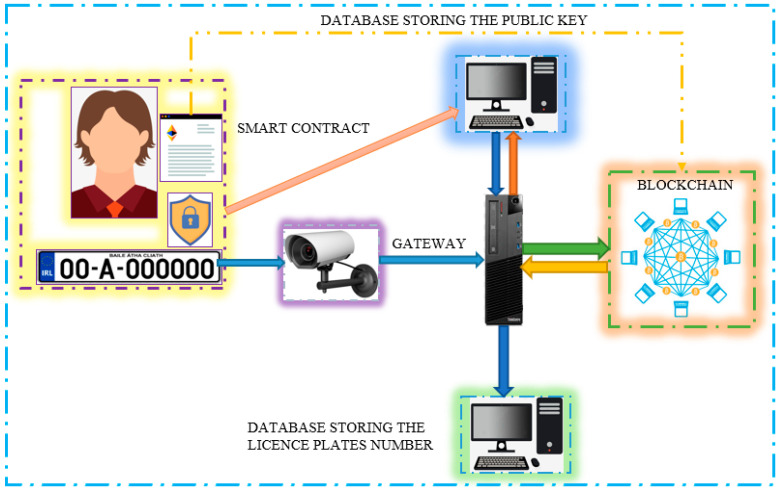
Architecture of LPR system.

Figure 3 shows the planned architecture for the LPR system. It has the smart contract that sends the signal to the user system, which is where the camera recognises the license plate and conducts the database search. These parts all communicate with one another via blockchain technology.

### 3.6. VANETS

This section will examine the current security service offerings provided by major VANETs. Although some specific mechanisms have been discussed, the design of any mechanism must consider usability difficulties. The emphasis in each part will be on various security requirements [25,30]. The comparison of Authenticated Routing for Ad Hoc Networks (ARAN), Secure and Efficient Ad hoc Distance Vector (SEAD), and Secure Message Transmission (SMT) is presented in Table 2.

- Identification Mechanism—The administration of vehicle-related identification is an intriguing element. The license plate immediately identifies the car in a unique way, unlike a traditional computer network where there is no centralised registration. In actuality, a legal authority and two manufacturers carry out the process. The manufacturer gives each car a unique vehicle identification number (VIN). On the other hand, the automobile license plate has been sought by the legal department, wherein each vehicle registration management domain is given a VIN that is utilised to specifically identify the purpose of producing a vehicle license plate.
- ARAN—All nodes in the server must have a public key certificate in order to use this technology’s public key encryption. A route discovery packet (RDP) that finds routes to all neighbours is broadcast by the source node. The communications that a node obtains from its neighbours are recorded by each node. When a neighbour receives the identical message, all subsequent communications are transmitted to them with their own logo and certificate. The first node reacts to the message when it reaches the destination.
- SEAD—The security of the new routing protocol was added to thwart attackers from creating more bizarre and wrong routing for any other node. Its foundation is destination-sequenced distance-vector routing. In place of more costly asymmetric encryption operations, this technique uses a one-way hash function. An initial node, established at random, chooses the one-way hash function.
- SMT—We also propose an end-to-end agreement and a secure messaging system. In order to do so, the source destinations must be associated with security. There is not a middle node for the encryption used. The source first identifies the first set of Active Path Sets (APS) for communication by using the current routing protocol. Once a set of the source APS was finished, each incoming message source was split into many portions, coded, and transported over various routes. The integrity and authentication of the source are verified using the MAC address that is carried by each dispersion sheet. Based on the APS failure or reception at the source node in various packets, rate the path. The source acknowledgement is sent together with a target-only verification acknowledgement.

**Figure 4:**
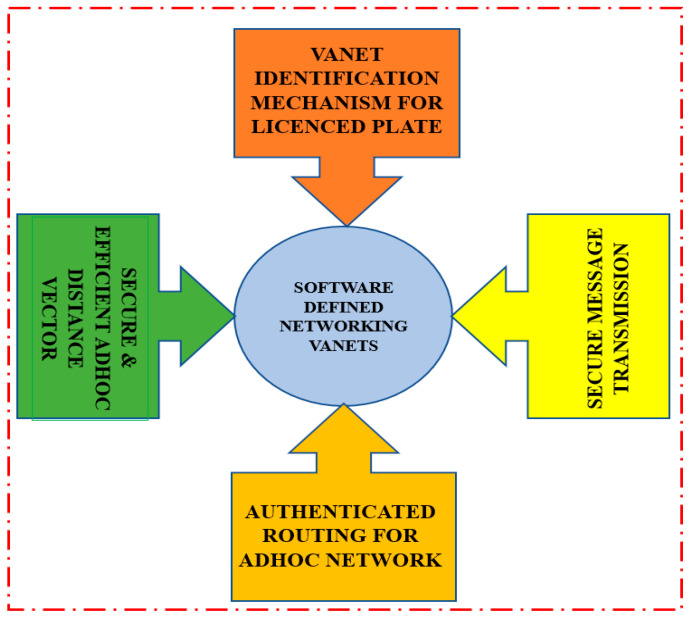
Simplified view of VANETS.

Figure 4 shows the simplified view of the VANETS, showing the Identification Mechanism, ARAN, SEAD, and Secure Message Transmission.

## 4. Methodology

This section describes the potential of integrating blockchain technology into ITS in smart cities in detail. ITS gathers data on traffic congestion, traffic management, and environmental changes, and it includes data from wired and wireless communications. LPR (License Plate Recognition) systems that use a range of strategies, including object detection, image processing, and pattern recognition, retrieve the license identifier. A real-time vision system has been developed using an efficient computing technique to track the tail lights of approaching vehicles and recognize motion parameters such as brakes, turns, and other actions. Blockchain technology has offered a decentralized data storage environment, transparency, and security to address the privacy concerns of IoV. The blockchain-based IoV system has been developed to facilitate vehicular data trading. The system uses smart contracts to manage the key functions and interfaces with the blockchain node.

### 4.1. Intelligent Transportation System Blockchain Model

A transportation system called ITS gathers and disseminates data on traffic congestion, traffic management, and environmental changes. Vehicle-to-vehicle (V2V) and vehicle-to-infrastructure (V2I) communication capabilities of ITS are extensive [31]. ITS also includes data from wired and wireless communications. While infrastructure-to-infrastructure communication typically uses wired technology, V2V and V2I frequently use wireless technology. Data collection for ITS tasks such as congestion monitoring, electronic toll collection, traffic signal cameras, traffic updates, and environmental forecasting uses the IoTs [31]. Authorities carry out the following tasks in an ITS system:

- Data gathering: IoTs are permanently installed in each ITS node to collect data from a variety of sources. Data concerning delays, location, journey time, monitoring, etc., are gathered by IoTs.
- Data transmission: Every node and vehicle in ITS prioritises real-time connectivity. The data is sent to a nearby data centre by vehicles. Data is supplied back to the affected cars as information following data analysis. Dedicated short-range communication is used for V2V communication, whereas continuous air interface is used for V2I communication at long and medium distances.
- Data analysis: The procedure of data cleaning, error rectification, data synthesis, and additional logical analysis starts after the data are received at the closest data centre. Both the current condition and future projections employ this analysed data.

**Figure 5:**
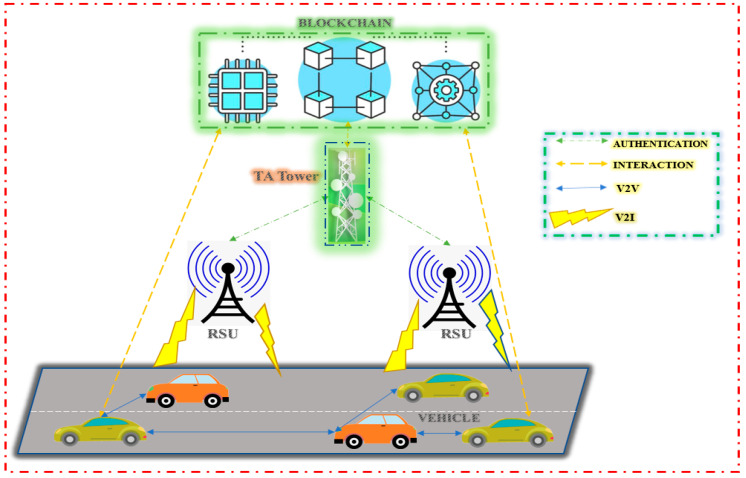
Model for intelligent transportation system network using V2V and V2I communication techniques.

The architecture of the network for intelligent transportation systems on blockchain is shown in Figure 5 below.

### 4.2. System for LPR

**Figure 6:**
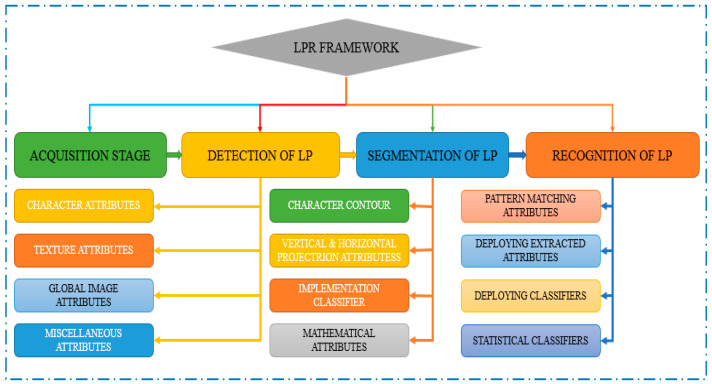
Basic classification of LPR framework.

The LP number on a car can be recognised using LPR systems, which are automated image processing tools. These systems relied on a single camera or many cameras that produced images that may be coloured, black and white, or infrared [32]. LPR systems use a range of strategies, including object detection, image processing, and pattern recognition, to retrieve the license identifier. Following the acquisition of the license plate number, it can be linked to information kept in databases, opening up the possibility for additional studies for a range of applications. Take electronic payment systems, traffic volume monitoring systems, surveillance systems, and vehicle tracking systems as illustrations. An LPR system recognises an LP’s number when it detects a vehicle, and this number is subsequently compared to vehicle database records of interest in criminal investigations. However, municipal organisations and regular people have questioned how well these techniques secure the private information included in the LP [32]. Figure 6 shows the proposed License Plate Recognition (LPR) framework, which consists of four processes. These processes are the acquisition stage, license plate detection, LP segmentation, and recognition of the LP. The acquisition stage involves capturing the license plate image through a camera mounted on a vehicle or a stationary object, such as a traffic light. Once the image is captured, the license plate detection process uses image processing techniques to locate the license plate area in the image. The LP segmentation process then extracts the characters or digits from the detected license plate area, and the recognition of the LP process identifies the alphanumeric characters on the license plate. This process typically converts the image of the characters into text that can be interpreted by a computer. The proposed LPR framework aims to improve the accuracy and speed of license plate recognition, which is crucial for traffic management and control. By automating the license plate recognition process, the proposed framework can reduce human error and enhance the overall efficiency of transportation systems.

### 4.3. License Plate Image Based Recognition Module

**Figure 7:**
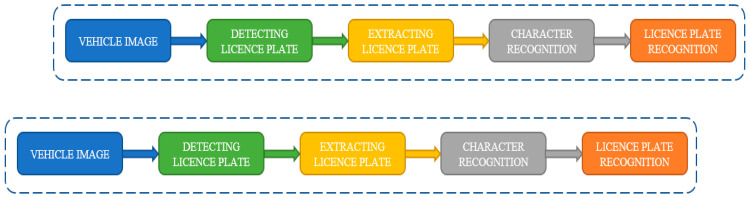
License plate detection and recognition.

It is insufficient to rely just on wireless sensor networks (WSN) and radio frequency identification (RFID)-based technologies to create ITS in smart cities. WSN and RFID-based standards are incompatible with the vast majority of on-road cars. Researchers are particularly interested in the area of image and video processing. A real-time vision system that can track the tail lights of approaching vehicles is developed using an efficient computing technique. Two different methods are used by the IVS picture mode to specifically identify an on-road vehicle. A real-time vision system that can track the tail lights of approaching vehicles and recognise motion parameters like brakes, turns, and other actions is developed [33,34] using a computationally efficient manner. A rolling shutter is replaced by a global shutter to guarantee that the images the camera captures are devoid of distortion or blur. The thing that needs to be photographed is moving, thus a slow or rolling shutter may produce a blurry image [33] that is useless for a license plate identification system. Figure 7 shows the license plate detection and recognition procedure step by step:

### 4.4. Blockchain Technology

Blockchain offered a decentralised data storage environment in addition to transparency and security. The block header and the body are the two parts that make up its structure. While transaction data are maintained in the block body, other metadata, such as the previous hash and timestamp, are kept in the block header. The following domains—consortial, private, and public—are the foundations for the various network access control techniques used by blockchain. To address the privacy concerns of the IoV, however, blockchain may offer a more dependable and scalable alternative [34]. In a blockchain-based IoV system, every node will have equal rights. They also have to follow the same rules. As a result, even if a node is excluded from the process, it will not have an impact on how the IoV works. Again, since it is an open and transparent system, there is no need to build up any trust with the vehicles. Each node will take part in the ITS system’s upkeep in this case. In order to solve privacy and security concerns and foster trust, the vehicle data management industry has embraced blockchain technology. In addition, the distributed data in the study was managed securely using blockchain in conjunction with an IoV network [35], in order to use a blockchain-powered framework called “DrivMan” to address the problems with trust management in the IoV system. New security and privacy issues have also been brought forth by this breakthrough. To facilitate vehicular data trading, however, BC technology is being included into the IoV.

### 4.5. Blockchain Based IoV System

A smart contract, which must be bundled into a transaction and broadcast to the blockchain network when the system is first started, implements the system’s key management function. It is necessary to document the contract’s address and interface information. When communicating with the blockchain node, the contract must send the appropriate contract address because only that address will allow the contract to carry out the intended function. For the system to find the elliptic curve E in the finite field or Galois Field (GF), Trusted Authorities (TA) must set the parameter L=(p,a,b,G,n) in order to construct the blockchain system GF(p). Table 3 lists a few of the key elements of the IoV system:

Key Management: The main issue that must be resolved in a cryptography-based application system is key management. The traditional IoV system allows the central authority entire control over controlling the storage and transmission costs associated with the binding of the vehicle identifier and public key. As more vehicles are added to the system, the main server may crash. If the data on the central server is changed without the administrator being made aware of it in time, the system will incur significant losses. Since the IoV system’s vehicles are travelling at great speeds, they must act swiftly. The central server is unable to address the problem of network congestion because of the lengthy connection distance [35].

(1) $$k_{i}=h(ID_{i}\;||\;r_{i})$$

where $k_{i}\;$is the public key for vehicle i, $ID_{i}\;$is the identifier for the vehicle, $r_{i}$ is a random number generated by the vehicle, and h() is a one-way hash function. This equation shows how the public key is derived from the vehicle identifier and a random number, ensuring that each key is unique to the vehicle and not easily guessable. In this way, vehicles can securely communicate with other entities in the IoV system without the risk of unauthorized access or tampering of data. Effective key management is crucial to the security and reliability of any cryptography-based system, and equations such as (1) are important tools in achieving this.

Key Revocation: The blockchain system uses a procedure called key revocation to identify malevolent individuals and revoke their public keys after analysing the behaviour of the cars. By using smart contracts to track malicious actions, the system develops a voting mechanism for malicious activity. The blockchain will keep the transactions that contain the vote results. The goal of key revocation is to identify rogue users within the system. The entire process functions as such: The roadside unit or blockchain node gathers the vehicle ID and malicious activities after receiving the distorted broadcast message from the malicious user, bundles them into a transaction, uploads them to the blockchain, and activates the vote record function in the smart contract. After receiving the vehicle communication, RSU obtains the car’s position data and utilises that data to determine whether the message has been significantly distorted.

(2) $$blc=C_{L}\left(L_{RSU},\;L_{V}\right)$$

The Equation in (2) is called blc, which stands for Blockchain Ledger Check. It is used as a tool to implement key revocation in the blockchain-based license plate recognition system. The goal of key revocation is to identify and revoke the public keys of malicious users within the system. The blc equation takes in two inputs, $L_{RSU}\;$and $L_{V}$, which represent the license plate number of the RSU and the vehicle, respectively. $C_{L}\;$is a function that takes in the license plate numbers and outputs the blc value.

In this scenario, the system will discard the data packet and then utilise the smart contract to check the vehicle for any recent instances of malicious behaviour before recording the findings. The blc equation is shown in Equation (1); if blc is false, the message has really been significantly damaged. Blc must be true for RSU to conclude that this communication is typical. The importance of key revocation in preserving system security cannot be overstated. To lower system risks and guarantee the system’s regular operation, the system must set realistic criteria.

**Figure 8:**
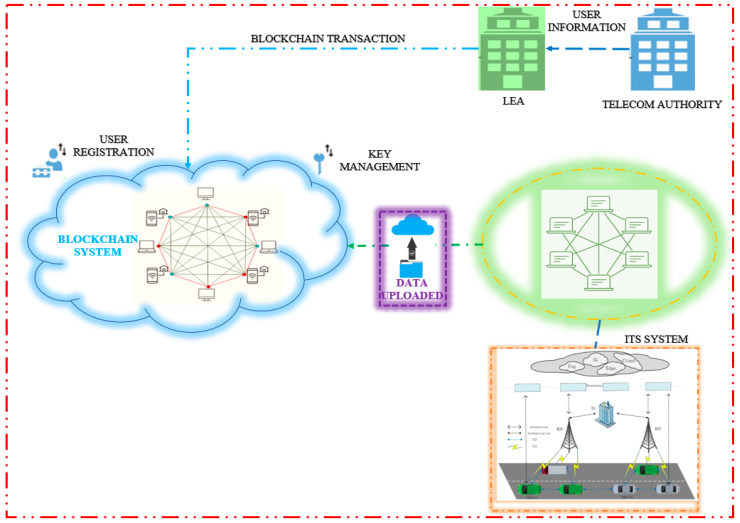
Blockchain-based IoV system.

The user’s private key and other public information are protected using the blockchain to prevent access. In the event of disputes, the blockchain system could keep the appropriate nodes liable. As shown in Figure 8, the LEA is also incorporated into the system to evaluate user- and TA-generated data before signing and transferring it to the public blockchain.

### 4.6. Module Description

The ITS architecture includes VANET as a key component. VANETs are also referred to as “intelligent transportation networks” at times. There must be some commercial apps that utilise VANETs before they may be deployed. User-based applications and safety-related applications make up the two types of VANET apps, which have the potential to be very important. Due to their lack of familiarity with one another, many vehicles in VANETs [36] are reluctant to divulge their personal information. By spreading misleading information, malicious cars can endanger the current network. The system needs data from a reliable source; thus the message must be true, believable, and unchangeable.

For the purpose of maintaining anonymity on the blockchain, the Lexicographic Merkle Tree (LMT) is utilised. The blockchain separates the real identity of the vehicle from its public key. For both new and dangerous cars, certificates are issued and revoked by the CA. CA [30,35] records every action it performs and stores it on the blockchain in order to maintain transparency. The authors offered a strategy for combating fake messages that includes the reputation system that the algorithm depends on to decide on the reputation. The V2I [36] authentication mechanism offered by the proposed system contributes to the dependability of CA. With the help of this model, harmful automobiles can be held accountable [37,38]. When compared to other systems, they offer security analysis that works effectively.

#### 4.6.1. User Based Application

These programmes are employed to improve traffic safety. The following subcategories can be applied to these applications.

- Internet Connectivity: People are constantly looking for ways to connect to the internet. Therefore, VANET offers consumers continuous access to the internet.
- Rapidly changing network topology: in a network topology that is rapidly changing, it will be faster to update the distribution to maximize on-the-road congestion and other signals, such as collisions and other vehicle, so that they can select their own alternative way.
- Frequent exchange of information: in order to collect data from other vehicles and roadside equipment, information is routinely shared, briefly activating VANET nodes.
- Wireless Communication—Communication across wireless networks: VANET is built for these conditions. Wireless connections allow nodes to communicate and exchange information. As a result, communication must take some security precautions into account.
- Unbounded network size: VANET can be built for the entire nation as one city or numerous cities. This implies that the network size of VANET is geographically limitless.
- Time Critical: in a VANET, the information must be delivered to the nodes within a predetermined window of time to allow them to make decisions and take necessary action.
- Better Physical Protection: The VANET nodes have greater physical security. As a result, VANET nodes are harder to physically compromise, which lessens the impact of an infrastructure attack.

#### 4.6.2. Safety Related Application

The adoption of these applications helps to make roads safer. The following additional categories can be applied to these applications:

- Co-operative Driving: the “curve signal cooperation and safe driving speed warning” and the “lane change warning system” [39] are two examples of continuous traffic-related warnings that the driver may get while driving.
- Collision Avoidance: Some studies claim that if drivers were given a warning just a fraction of a second before colliding, 60% of accidents might be avoided. Getting a warning message in time can help a driver avoid an accident.
- Traffic optimization: You can reduce the amount of time it takes to transmit by making the most of traffic jams and other signals like accidents and other cars so that they can choose their own alternative routes.

#### 4.6.3. Communication Related Applications

Some of the applications are related to communication [40] energy [41], sensor [42,43] and power splitting [44].

## 5. Results

This section discusses the use of blockchain technology for LPR in the transportation industry. For this LPR project, the Application Programming Interface (API) was utilized, which offers the ability to locate objects in images and videos. However, only text that adheres to the license plate format is accepted. Therefore, checks for license plate pattern filtering were introduced. The character identification stage, which is part of the vehicle LPR framework, is where the extracted characters are identified by displaying the anticipated license plate numbers of the input vehicle LP pictures as the output. The number of the LP is determined in large part by this stage.

The RSUs, TDs, and various trust authorities form the blockchain. New identities for automobiles are created by a number of trust bodies. The $TA_{1}$ node uploads transaction data to the blockchain after being chosen by a proper voting system to serve as an accounting node. The notation used in this section is displayed in Table 4 below:

### 5.1. Registration of the Vehicle License Plate

In this line of work, the registration of a car is completed in two steps. First, the TA must create an ECC public–private key pair for the vehicle, namely $PV_{i}$ and $SK_{V_{i}}$, as well as a candidate transaction set that is ready for upload. Second, the RSU produces a vehicle pseudo ID. As indicated in Table 5, the TA creates a public–private key pair for Vi:

Table 5 details the registration process steps. The $VID_{i}$ provided by T D is presented by the cars when they register with TA for the first time. Using a specific consensus procedure, the system’s supervisory node (T D) must choose a node in charge of the vehicle’s registration. In this context, it is assumed that T A1 is only a temporary authoritative node selected for this registration.

$TA_{1}$ verifies the $VID_{i}$ and selects an integer b∈Zq as the private key of the vehicle, namely, $SK_{V_{i}}=b$ and generates a public key $P_{V_{i}}$, where $P_{V_{i}}=b\cdot P.\;TA_{1}$ sends $\langle b,P_{V_{i}},H\left(Sig\right),Sig,TA_{1}\rangle \;$to the vehicle via a secure channel while simultaneously creating a partial transaction set that is waiting to be uploaded that contains the genuine IDs of numerous vehicles. It is important to note that the public key certificates are encrypted with the public key of the $TA_{1}$ and stored in the partial transaction set as ciphertext.

### 5.2. Transaction to Blockchain

**Figure 9:**
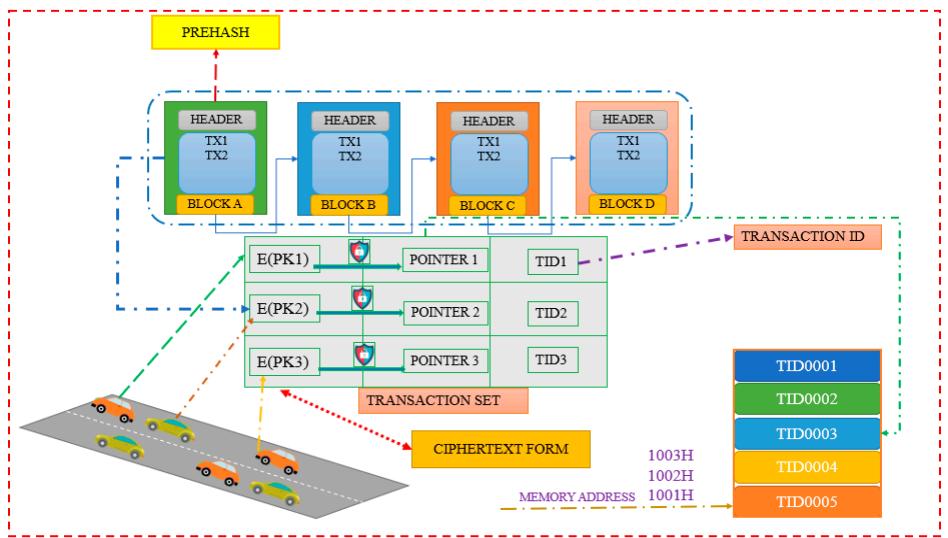
Blockchain transaction format.

After transmitting the pseudo ID to the car, the $RSU_{1}$ will convey a pointer to the memory location of the $PID_{i}$, known as $POINTER\;PID_{i}$, to the $TA_{1}$ and send the $PID_{i}$ (pseudo ID of a vehicle) generated for this vehicle to the trusted cloud server. The partial transaction that was previously waiting to be uploaded is where the pointer is recorded by $TA_{1}$. The $TA_{1}$ generates a whole transaction set at the same time and uploads it to the blockchain. A public key certificate encrypted with the public key of $T_{1}$ is included in every transaction in blockchain, along with a pointer and a transaction ID, as shown in Figure 8. The vehicle’s registration information creates a transaction with the transaction ID $TID_{j}$, which is specifically identifiable. A vehicle can be identified by searching for its license plate number in blockchain records, using the transaction ID. The blockchain transaction format is illustrated in Figure 9.

### 5.3. Authentication between RSU and Vehicle

The vehicle $V_{i}$ exits the $RSU_{1}$-covered area and enters an area under $RSU_{2}$. The roadside unit and vehicle must successfully perform anonymous authentication. Table 6 breaks down the authentication procedure into four steps:

### 5.4. Privacy and Security Analysis

In the registration, the vehicle calculates message $M_{1}=a\cdot PV_{i}\cdot R_{1}$, where a is the private key of the vehicle. $RSU_{1}$ calculates messages $M_{2}=b\cdot PR_{1}\cdot \;R_{1}$ and M3 = R1 · M_1, where b is the private key of $RSU_{1}$. Mutual authentication is completed between two communication participants by confirming that $M_{2}\cdot R_{1}\;and\;R_{2}\cdot M_{1}$ are equal. Unless the attacker has access to their private key, messages encrypted with their public key cannot be deciphered. An elliptic curve discrete logarithm problem (ECDLP) issue specifically arises during the private key acquisition phase. Thus, our plan satisfies confidentiality requirements. In our system, there is no single point of failure. First off, a trustworthy ledger with authority is jointly maintained by a number of TAs and RSUs. For cars and RSUs, each TA is in charge of dispersing public–private key pairs. Second, the RSU creates a fictitious ID for the vehicle in our scheme, weakening the permissions of the authoritative node TA. A blockchain with authority can be used to guarantee distributed features. By delivering the identical packets again, the RSU can trick the system. However, it is only possible to know the random numbers $R_{1},\;R_{2}$, by themselves. It can prevent the replay attack from decoding the car’s private key by making sure that the request and reply between the car and the RSU do not use a fixed connection. The vehicle must first be CA-authenticated before authentication.

## 6. Discussion

In the area of intelligent transportation, the “IoV” is an important application that has generated a lot of interest among academics. Privacy protection is a top priority for the IoV system. The task of transportation information systems is to process and disseminate data about traffic, traffic management, and environmental changes. While infrastructure-to-infrastructure connections are normally made via conventional techniques, wireless technology is typically used for V2I and V2V. With the help of the IoT, ITS collects data for traffic signal cameras, congestion detection, electronic toll collection, traffic updates, and weather forecasts.

This study suggests a blockchain-based privacy protection system for the IoV that uses LPR. It introduces an anonymous communication technology between vehicles and service providers, ensures the security registration process, successful identity management scheme, two-way identity verification based on blockchain, a mutual authentication algorithm based on random numbers, and multiple authentications based on blockchain in order to provide the privacy of security information. Additionally, it is said that the gateway in charge of regulating all communications receives a picture of a license plate taken by an LPR equipment. When a user wants a license plate, a system handles the registration directly on the blockchain, skipping the gateway.

Private and public keys for the user are also generated at this point. Only the authorised addresses are able to change the privacy settings of a smart contract after it has been registered in the private chain. The number on a vehicle’s license plate may also be recognised by LPR systems, which employ automatic image processing. A vehicle’s license plate number can be located via an LPR system, and this information is then compared to relevant vehicle database entries in criminal investigations. By retaining car data that is not required for investigations, concerns regarding the privacy of common people have been raised. The project team highlighted key management as the main problem that needs to be fixed in the blockchain-based application system. In the traditional IoV system, the coupling of the vehicle identification and the public key is completely under the jurisdiction of the central authority. As the number of vehicles in the system increases, the database controller could crash. The blockchain technology can identify malicious people and revoke their public keys by analysing vehicle behaviour. The process is referred to as key revocation. To keep track of unethical behaviour, the system creates a voting mechanism for malevolent behaviour that runs on the blockchain. The transactions that are enclosed with the results of the voting will be stored on the blockchain.

There can be some limitations of this study. Firstly, the proposed blockchain-based system may not be immune to all types of attacks, and the authors have not evaluated the system’s resilience to potential attacks. Secondly, the paper mainly focuses on privacy and security concerns in LPR within the context of the IoV, without examining other potential ethical and legal implications of the system. Thirdly, the experiments conducted in this study are not extensive enough to fully evaluate the proposed model’s performance and its generalizability to different scenarios. Additionally, this paper assumes that all vehicles are equipped with the necessary technology for license plate recognition, which may not be feasible or cost-effective in the real world. The paper also does not address the potential impact of the proposed system on the environment and resource consumption, which can be a significant concern for large-scale transportation systems. Finally, the proposed system relies on the availability and reliability of the blockchain technology, which may not be available or accessible in all regions or countries. Overall, while the proposed system shows promising results, more extensive research and evaluation are needed to address these limitations and fully realize the potential of blockchain-based LPR in the IoV.

## 7. Conclusions

Preserving privacy is the highest priority for the decentralized network used in this ITS study. The strategy is based on blockchain technology, which enables consensus among a decentralized network, even with problematic nodes. The main players in this scenario are vehicle license plates and a centralised organisation in charge of blockchain storage and trust computation. The connection between the vehicle’s license plate and the host computer is only done in an encrypted manner during the decision phase, which involves all of the license plates of vehicles in the same region. Based on existing methods, the detection, segmentation, and recognition of the plates have been the main areas of focus for the LPR framework. This study presents an LPR-based future projection and discusses some potential difficulties in this area.

A study recommends a blockchain-based approach for IoV privacy security that makes use of LPR. Between automobiles and service providers, it introduces anonymous communication technologies. A system handles the registration of a user’s license plate directly on the blockchain, avoiding the gateway. The first issue that needs to be resolved in the blockchain-based application system, according to the project team, is key management. The database controller may crash as the number of vehicles in the system rises. The programme develops a voting process for malicious behaviour that utilises the blockchain. Researchers have shown a large amount of interest in the IoV as a significant application in the field of intelligent transportation. Protection of privacy is given top attention by the IoV system. 

The IoV privacy protection system presented in this work is based on blockchain technology. It introduces secure user registration, efficient key management schema, two-way blockchain authentication, a key agreement technique based on random numbers, and anonymous communication technology between the vehicle and service provider to ensure the privacy of security information. The system makes use of the decentralised function of the blockchain to get around the central failure problem. The approach suggested in this paper outperforms the previous method in terms of communication efficiency and communication security. However, the system’s existing proof-of-work-based consensus mechanism will suck up computer power, and the key exchange method will put some storage under pressure.

In the future, the intention is to develop a more effective consensus method than the proof-of-work algorithm and a communication data structure that is more efficient in order to reduce system costs, increase overall efficiency, and improve the user experience. To further support real-time communication, we will keep researching ways to shorten the time needed to query public keys.

## Figures and Tables

**Table 1:** List of past paper references with methodology used and results.

Authors	Methodology	Experimental Parameters/Dataset	Libraries/Platform Implementation	Outcome/Results	Main Focus
[1]	FedLPR, LPR	Dataset includes nearly 32,000 high-quality images. 2000 licensed plate images, 2000 users in the application	LPR Model, Tensor Flow Framework, Snapdragon Processor.	Solving the User Privacy. For environment 1: recognition is around 94% and Detection is around 100%.	Traffic Management, User Privacy, LPR
[2]	Statistical Methods, ANN.	License Plate Extraction Algorithm, NRC, NFC, TNFC, FNFC	Intel Pentium Core 2 Duo System Used for Implementation.	NRC: Set 1–967, Set 2–867 NFC: Set 1–241, Set 2–108	Gray-scale Images, Automatic LPR
[3]	LR and Neural Networks, Blockchain, Edge Computing, Maxchine Learning	1000 training records/1000 testing records	Raspberry Pi, Merkle tree, Anchoring Protocol	LR model is 144 bytes and the number of training rounds is 100.	Blockchain-based FL, LPR
[4]	IVS Zigbee Protocol Stack, Data Collection Cloud	Image passes via hierarchy of image manipulation phases having a shutter speed of at least 1/1000.	Wireless Sensor Networks, VANETS, RFID, ITS, IVS, Decision Module, Micocontroller	Implementing full-scale Intelligent Transportation System	Multifaceted Vigilare System for ITS
[11]	Deep Learning, ML, Blockchain, Gradient Descent, RMSProp, FedAdaGrad Algorithm	The BraTS 2018 dataset included roughly 300 people’s MRI images. MRI Data Collection.	FEDF Framework, CIFAR-10, Python Library, LEAF Platform	Improved security and handling of data	FL, LPR
[5]	ML, DL, Mobile Edge Computing, Computer Vision, DNN, Natural Language Processing, CNN Model, Blockchain.	Flickr-AES dataset, LoAdaBoost FedAvg algorithm, MNIST dataset.	LEAF, SyftTensor, Pytorch Tensor, PointTensor, FATE Open Source. CIFAR-10	Attack Success rate for Baseline is 0%, for Attack 1, it is 35% and for Attack 2, it is 96%.	FL in Mobile Edge Networks
[6]	VLSTM, Variational Bayes, Feature Representation, F1, AUC, and FAR scores, Naïve Bayes, RF, AdaBoost	Dataset named UNSW-NB15, total feature dimensions reach to 196. 9 types of Anomalies in the Dataset	IXIA PerfectStrom tool, Python 3.6, LSTM Encoder Module	Designing VLSTM learning model. Precision. F1–0.907, FAR–0.117, AUC–0.895	LSTM Enhanced Anomaly Detection, LPR
[7]	Blockchain, DSRC, WAVE, VANET, Bitcoin, P2P Network	IV-TP Dataset	Merkle Tree, Hash function (SHA-256)	Unique ID (Trust Bit), Handshake Layer, Reward Layer. Proof of driving verified and validates the communication network for vehicles. Proposed vehicle information sharing architecture for real-time traffic data.	Intelligent vehicle, LPR

**Table 2:** Comparison table for ARAN, SMT, and SEAD.

Solution	Technology Used	Attacks Covered	Security Requirements
SMT	Message Authentication Code (MAC) Address	Information Disclosure	Authentication
ARAN	Cryptographic Certificate	Replay Attack False Warning	Authentication Message Integrity
SEAD	One-way Hash Function	Routing Attack Resource Consumption	Availability Authentication

**Table 3:** Components of IoV system.

Components	Description
Roadside Unit (RSU)	RSUs are distributed nodes that gather regionally trained models to train the aggregated model at the global level.
Certification Authority (CA)	Cypher suites are made available by the certifying authority to guarantee secure data transfer in the communication network. The centralised certification authority handles the IoV system registration of cars and RSUs.
Vehicles	Automobiles are dynamic, mobile edge computing devices. There are two different kinds of cars: common edge vehicles and group representative vehicles chosen by the RSU.

**Table 4:** Notation.

Notation	Meaning
R	Random numbers
E ()	Encrypt
POINTER	A pointer to the storage location of Pseudo ID
TIDj ( )	Transaction ID of j th transaction
Ti()	Timestamp
TXi ()	Candidate transaction set
PRi	The public key of i th RSU
SKvi	The private key of vehicle
PVi	The public key of vehicle

**Table 5:** Registration of the vehicle license plate.

*1*. $V_{i}\to \;TA_{1}:\;\langle \;VID_{i}\;||\;$***Other***〉
*2*. $TA_{1}\;\to \;VID_{i}\;:\;\langle \;Verify\;\left(VID_{i}\right)\;\rangle$
*3*. $TA_{1}\;\to \;V_{i}$***:***$\langle \;d\;|\left|\;P_{V_{i}}\right||\;Sig\;\{\;H\;(P_{V_{i}}\;||\;d)\}\;||\;T_{i}$〉

**Table 6:** Authentication process between RSU and vehicle ID or number plate.

**Step 1**	The vehicle sends an authentication message Mi including PIDi, $Cert_{PID_{i}}$, $H(Cert_{PID_{i}})$, a timestamp T_2_ and a transaction ID encrypted with the public key of ***RSU*_2_** namely <$TID_{j}$> to ***RSU*_2_**.
**Step 2**	After receiving the message Mi, the $RSU_{2}$ decrypts the message ***Mi*** by using its private key $a_{2}$ and gets the PIDi transaction ID ($TID_{j}$), and timestamp T2. The $RSU_{2}$ can verify the legality of the vehicle by querying the blockchain using $TID_{j}$.
**Step 3**	Based on the transaction ID provided by the TA**_1_**, the $RSU_{2}$ can quickly know identity information of the vehicle by visiting transaction information instead of traversing the entire blockchain system. Firstly, through the transaction information recorded in the blockchain, the $RSU_{2}$ determines whether the transaction information corresponding to the $TID_{j}$ exists.
**Step 4**	Once the legality of vehicle identity is verified, $RSU_{2}$ can provide the corresponding service to it.

## Data Availability

Data are available on request.
